# ‘A radical operation’ – a thematic analysis of newspaper framing of bariatric surgery in adolescents

**DOI:** 10.1186/s12889-023-15366-8

**Published:** 2023-03-07

**Authors:** Sander Lefere, Kato Verghote, Ruth De Bruyne, Veerle Provoost, Priya P. Satalkar

**Affiliations:** 1 Hepatology Research Unit, Department Internal Medicine and Pediatrics, Ghent University, Ghent, Belgium; 2 Liver Research Center Ghent, Ghent University, Ghent, Belgium; 3 Department Moral Sciences and Empirical (Bio) Ethics Research, Bioethics Institute Ghent, Ghent University, Ghent, Belgium; 4 Pediatric Gastroenterology, Hepatology and Nutrition, Department Internal Medicine and Pediatrics, Ghent University, Ghent, Belgium

**Keywords:** Childhood obesity, News media, Sensationalism, Health disparities, Stigma

## Abstract

**Background:**

Obesity in adolescents is a growing public health issue. Bariatric surgery is an effective, yet controversial treatment option for adolescents. The moral acceptability of this procedure by health-care professionals as well as the general public can be influenced by its portrayal in the news media. Our objective was to analyze how newspaper articles portrayed adolescent bariatric surgery, with attention to the language used and moral arguments made.

**Methods:**

Using an inductive thematic analysis approach, we analyzed 26 UK and 12 US newspaper articles (2014–2022) on adolescent bariatric surgery for implicit or explicit moral evaluations and use of normative language. Coding was performed after immersive reading, assisted by NVivo. Themes were identified and refined iteratively through consecutive auditing cycles to enrich the depth and rigor of our analysis.

**Results:**

The major themes identified related to (1) defining the burden of adolescent obesity, (2) sparking moral outrage, (3) sensation-seeking, and (4) raising ethical issues. The articles employed moral language, specifically non-neutral and negative discourse regarding surgery. Blame was attributed to adolescents or their parents. Sensationalist wording often reinforced the normative content, drawing the attention of the reader and contributing to stigmatization of adolescents with severe obesity as lacking will power and being lazy. Further moral issues that stood out were the challenges in obtaining an informed consent, and the unequal access to surgery for socially disadvantaged groups.

**Conclusions:**

Our findings provide insights into how adolescent bariatric surgery is represented in the print news media. Despite frequent citing of experts and studies on the efficacy, safety and unmet need for bariatric surgery, obesity and surgery in adolescents are often stigmatized and sensationalized, with (prospective) patients depicted as looking for an easy way out in the form of a solution brought by others (health systems, society, tax payers). This may increase the stigma surrounding adolescent obesity, and therefore limit the acceptability of specific treatments such as bariatric surgery.

**Supplementary Information:**

The online version contains supplementary material available at 10.1186/s12889-023-15366-8.

## Introduction

The number of adolescents with obesity has doubled over the last decades, now affecting one in every five in the United States (US). Concurrently, the prevalence of severe obesity (defined as a BMI ≥ 35 kg/m^2^ or ≥ 120% of the age- and sex-adjusted 95^th^ BMI percentile) has greatly increased as well [1]. Contrary to the idea of ‘growing out of obesity’, the majority of obese children and adolescents become obese adults [2]. Adolescents with obesity are at risk of metabolic and cardiovascular comorbidities, including type 2 diabetes, hypertension and non-alcoholic fatty liver disease [3, 4]. In this setting, timely treatment is of importance. Lifestyle management is the first line of treatment and can reduce comorbidities, yet especially in adolescents with severe obesity, the long-term efficacy is rather limited [5].

Over the last two decades, bariatric surgery has emerged as an effective treatment option for adolescents with severe obesity [6]. A population-based US study found that over 14.000 bariatric procedures were performed in patients ≤ 20 years of age between 2005 and 2014, although the majority were performed in young adults (mean age 18.6 years) [7]. The rate of bariatric surgeries in the US has increased gradually over time to 331 cases per 1 million adolescents [8]. Longitudinal studies have reported BMI reductions of 13–16 kg/m^2^ [9]. In comparison to adults, weight loss outcomes are similar in adolescents, yet resolution of comorbidities was attained in a significantly higher proportion, suggesting that early surgical intervention might be preferred [5].

Nevertheless, bariatric surgery in adolescence remains controversial and presents physiological and psychological challenges during this period in the patient’s life, when they are transitioning to adulthood and emerging independence. Even when weight loss goals are met, most patients remain overweight or obese, which might have negative psychological consequences, especially in those with unrealistic expectations regarding weight loss [10]. The lack of long-term outcome data adds to the uncertainty of whom to treat surgically.

One reason for the controversial nature of bariatric surgery in adolescents is the fact that obesity, childhood obesity in particular, has social and moral connotations attached to them. People with obesity are often portrayed as neglecting their own health, or failing to eat healthy. Parents of children with severe obesity are judged to be ignorant, irresponsible, or even bad parents [11]. Given that obesity is correlated with a low socioeconomic status or belonging to a minority, blaming people with obesity may serve to reinforce social inequalities [12]. Framing obesity as an illness might limit blame by suggesting a biological or genetic determination, yet contribute to stigmatizing people with obesity as diseased [12]. A 2001 survey revealed that 65% of Americans agreed that obesity is at least partially the result of a ‘lack of willpower’ [13]. These views are important since research indicates that support for specific health policies is lower if obesity is seen as a personal issue [13, 14]. Furthermore, surgery in adolescents poses moral challenges concerning informed consent or assent, autonomy, and voluntariness [15].

News media are a major source of health information for the general public, and can potentially influence how health problems are viewed in society, increase stigmatization, and affect support for specific treatment options, prevention strategies and public health policies [16, 17]. Although more research is needed about the specific ways in which media frames influence the general public’s opinion and health policy debates, there is increasing acceptance that insight into these matters is crucial for advancing these debates [18]. Bariatric surgery in adolescents has gained significant coverage over the last decade, including articles which cite health-care professionals (HCPs), figures of authority to whom the general public might be attentive. Therefore, how adolescent bariatric surgery is framed in the news media, especially those that reach a wide audience, such as high-volume newspapers, might conceivably shape the public opinion on this issue, including moral judgments.

Our objective was to analyze how newspaper articles portrayed adolescent bariatric surgery, with attention to the language used and moral arguments made, as to our knowledge, this had not been investigated so far. We aimed to address this gap by performing qualitative thematic analysis to analyze, interpret and report moral judgement in media framing of adolescent bariatric surgery within the newspaper dataset [19].

## Methods

### Study site

We selected newspapers based in the UK and US. These countries are representative of the Anglo-Saxon world, yet have distinct socio-economic and healthcare contexts that might impact how the topic is framed in the media. Specifically, in the UK, resources for healthcare interventions are allocated through the National Health Service (NHS), whereas in the US the publicly financed Medicare and Medicaid health coverage coexists with privately financed coverage, which is unaffordable for many Americans. In both countries, pediatric obesity rates are high, and adolescent bariatric surgery has been available for some time. Consequently, obesity and bariatric surgery are being discussed not only in the scientific community but also in the national and regional media.

### Sampling

Articles were obtained through the LexisUni search engine of international media. All newspapers from the US and UK were included to obtain diversity in scope, readership and publisher’s ideological orientation. We searched for articles containing keywords referring to both bariatric surgery (‘bariatric surgery’, ‘weight loss surgery’, ‘sleeve gastrectomy’, ‘gastric sleeve’, ‘gastric bypass’, ‘gastric banding’) and adolescence (‘adolescence’, ‘adolescent*’, ‘teen*’, ‘teenager*’, ‘young adult’). Given that bariatric surgery in adolescents is relatively novel, we restricted our search to articles published from January 1, 2014 to February 28, 2022. Filters for articles in English and in the category ‘Medicine & Health’ were applied.

### Data collection

**Fig. 1:**
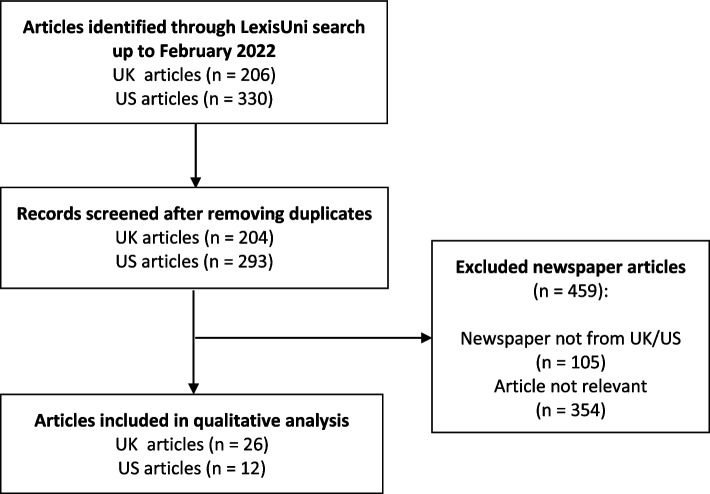
PRISMA Flowchart of the study selection process

The search strategy yielded 537 articles (205 UK and 332 US newspaper articles), which were downloaded in Word format. Duplicates and articles reprinted in newspapers based in countries other than the US or the UK were excluded. Selection criteria were a focus on adolescent bariatric surgery and relevance for an analysis of media framing; i.e. articles limited to describing the results of a scientific study on adolescent surgery were excluded. Given the broad search, most articles were not relevant for our purpose. After assessment of all articles by the first author, 38 articles (26 from the UK and 12 from the US) were included (Fig. 1). The list of all included articles is presented in Supplementary Table 1.

### Data analysis

Publication trends over time and according to media outlet were assessed to provide background information. Thematic analysis was performed [20] with a particular focus on the use of normative language and moral evaluations of adolescent bariatric surgery. The selected articles were read and re-read to get familiarized with the content. The articles were then imported into NVivo (v.1.6.1) by the first author, for inductive coding according to the methodology proposed by Braun and Clarke [19, 21]. Coding was discussed with a second author (PS). The coding was gradually abstracted to identify potential themes, which were evaluated and refined by the co-authors in a collaborative auditing cycle [22]. Consecutive auditing cycles were organized to enrich the depth and rigor of our analysis and prevent the final analysis from being a mere reflection of the first author’s subjective interpretation of the data.

### Ethics

The newspaper articles and data used for this study are publicly available and have been specifically published to reach a wider (non-scientific) audience. Personal stories of adolescents and their family, sometimes under a pseudonym, were published in these newspaper articles with consent of those involved. Moreover, personal data (age, names, location, disease course, treatment) were not directly quoted or included in our analysis. We therefore judged that our study did not invade their privacy. Similarly, the names and affiliations of HCPs quoted in the articles did not feature in our analysis. Quotation marks or bold text for emphasis within the quotes were adopted from the primary source and were not added by the authors.

As this was not feasible for practical reasons (e.g. journalists might no longer work at the newspaper, journalists might not have access to the (updated) contact information of their sources or are unable to share this for confidentiality reasons), we did not seek consent from either the journalists or the patients, their parents or HCPs portrayed in the newspaper articles. Ethical approval was obtained from the Ghent University Faculty of Arts and Philosophy Ethics committee (Nr. 2022/11).

## Results

**Fig. 2:**
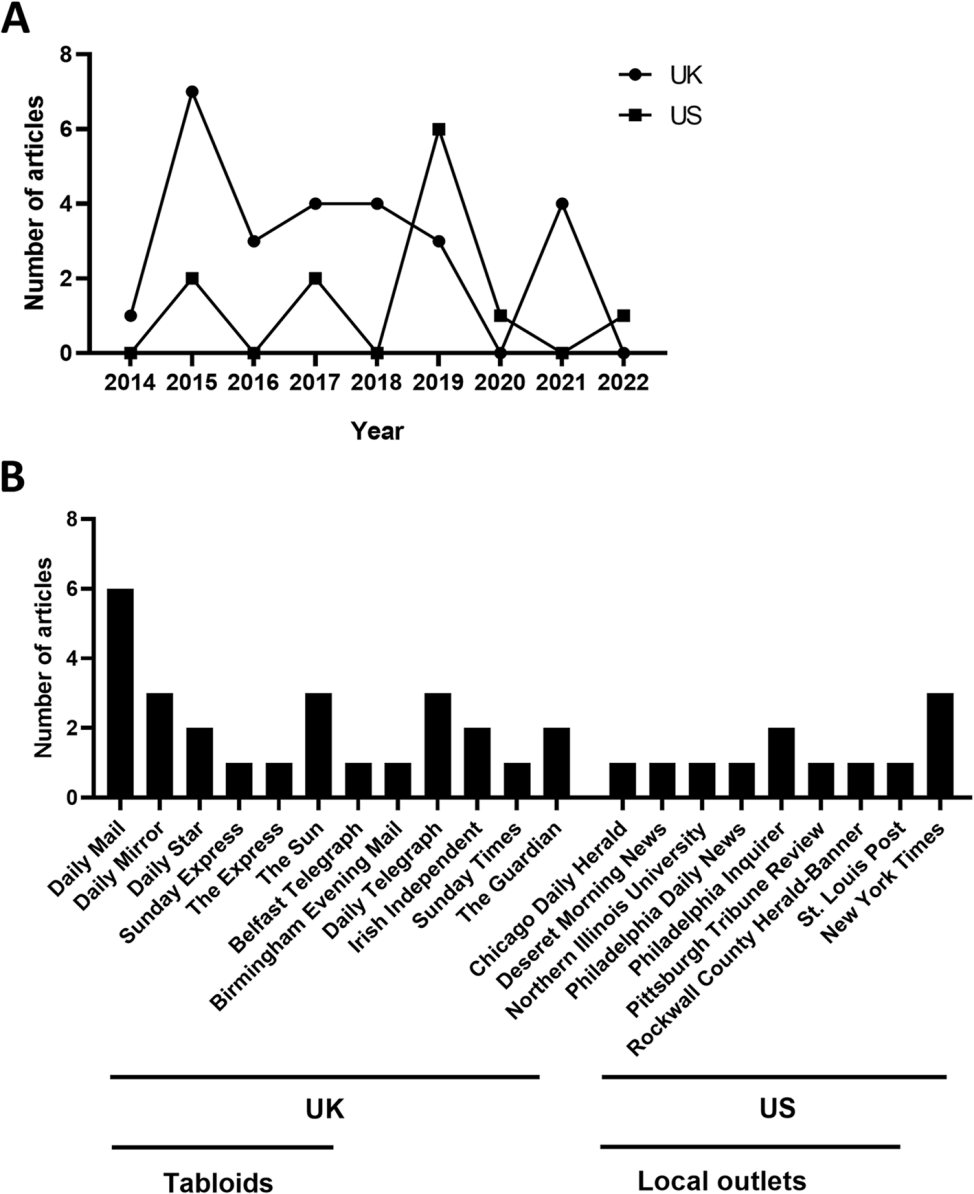
**A** Number of included UK and US articles published per calender year. **B** Breakdown of number of newspaper articles according to news publisher

The number of articles published each year varied widely, with a peak in 2015 (Fig. 2A). The number of articles published in US newspapers was lower than in UK newspapers in every year, except for 2019, when several US news sources published articles built on findings from a scientific study. In the UK, most articles (62%) were published by national tabloids, whereas the majority of US coverage was in local newspapers (75%) (Fig. 2B).

Four main themes were identified through thematic analysis, (1) defining the burden of adolescent obesity, (2) sparking moral outrage, (3) sensation-seeking, and (4) raising ethical issues. Several quotations are presented within the text, and additional quotations for each theme are included in Table 1.

**Table 1:** Structure of themes and subthemes with additional representative quotes not included in the main text

Theme	Subtheme	Representative quotes
**Defining the burden of adolescent obesity**	Statistics	“Officials at the World Health Organization (WHO) have warned that childhood obesity is one of the most serious global health challenges. Almost a third of UK pupils are already overweight by the age of 11.”
	Causes of obesity	“He says that the rise in obesity is a "devastating" reflection of today's lifestyles: There is a substantial proportion of teens out there who have barely had a healthy meal in their life, are living couch-potato lifestyles and are spending hours playing computer games. […]"
		“Surgery, of course, doesn't get to the root cause of why so many young Britons get fat in the first place: the proliferation of fast food outlets in deprived areas, sugary drinks and high-fat, high-sugar foods, poverty, fall-off in physical education in schools, the rise of a sedentary lifestyle and the allure of computer games and laptops."
		“Furthermore, in predominantly Black and Latino North Philadelphia, almost three-fourths of the children were overweight.”
**Sparking moral outrage**	Stigmatizing obesity	“She admits she felt "some disdain for moms who let their kids drink soda or eat fast food" and "sometimes looked down on the moms of fat kids". There was no junk food in her house. Her kids drank only water and ate three healthy meals
	Who’s to blame?	“I think the parents have to be blamed for overweight children, but they have to be helped. Although this is difficult for them—and we can understand chastising children and stopping them doing what they want is difficult—I'm afraid that's their responsibility. It comes with being a parent."
	Surgery is a ‘quick fix’	“Experts worry that teens are opting for quick-fix operations such as gastric bypass, gastric bands and gastric balloons instead of changing their diet and doing more exercise.”
		“One of the criticisms of weight-loss surgery is that it doesn't necessarily promote healthy eating: It's amazing what you can liquidise, Dr. XXX says”
**Sensation-seeking**	Headlines	“FAT KIDS OP PLEA—Overweight teenagers must have weight-loss surgery to stop 'obesity apocalypse', leading doctor claims"
	Personal stories	“A teenager who watched her obese mother die of a heart attack has undergone weight loss surgery at 16 to avoid the same fate. XXX, was just a toddler when her mother YYY, suffered a heart attack at the family's home in ZZZ. Then aged three, XXX watched in horror as her mother YYY collapsed in front of her in 2004. YYY, who weighed around 300lbs, knocked over XXX toy kitchen as she fell to the floor and had passed away by the time paramedics brought her to hospital. Terrified she would also die young, the teenager—who weighed 275lbs (19st 6lbs)—had a vertical sleeve gastrectomy at 16, and has since lost 100lbs."
	‘Radical’ surgery	“TEENAGERS as young as 16 have undergone radical NHS-funded weight loss surgery in Birmingham, worrying new figures show."
**Raising ethical issues**	Ability to give consent	“Even operating on teenagers raises issues that may not apply to adults. Can a miserable adolescent, for example, really can give informed consent to such a drastic, life-changing operation? And can a teenager be expected to commit to following the very restricted diet required after the surgery, not to mention taking the needed vitamins and minerals? Are they prepared to do that for the rest of their lives?”
	Unmet need and disparity in access to care	“Only 0.2 per cent of people who meet the official criteria—that is, having a BMI over 30 with diabetes that is not adequately controlled by medication, or having a BMI over 40 regardless of diabetes—get offered bariatric surgery. I would like that changed to it being offered to more people who at least fit the criteria as drawn up by the National Institute for Health and Care Excellence"
	Who should pay for surgery?	“The controversial operation, […], costs the NHS about £6,000 a time. […] Urgent action is needed and unless it happens the problem of obesity and related health costs will bankrupt the NHS.”

### Defining the burden of adolescent obesity

The first theme relates to a general discussion on adolescent obesity, focusing on obesity statistics and the causes of obesity. Although not strictly normative in nature, the undertone was not neutral, indicating a moral evaluation of the current situation or judgment on the lifestyle responsible for obesity.

#### Statistics

Articles often cited various statistical sources to underpin the growing problem of childhood obesity: *"The new statistics come from the annual Health Survey for England, which has been running since 1991 and involves interviewing and measuring 8,000 adults and 2,000 children every year. […] a "staggering" one in four teenagers was clinically obese by the age of 15.”* Descriptions similar to this one of a ‘staggering’ number of obese teenagers also featured in other articles, which regularly described the problem as an epidemic or a crisis: “*[…] childhood obesity is a crisis already engulfing the UK.”* In other cases, obesity was negatively framed in more explicit statements: “*[…] the nation's fattest children had become even heavier in recent years, sparking fears of a costly obesity timebomb. […] Unfortunately those children who are already obese are getting even fatter*”. The metaphor of obesity as an ‘exploding problem’ was recurrent in our dataset, for instance *“There has been an absolute explosion in the extreme end of obesity in kids.”*

#### Causes of obesity

In conjunction with obesity statistics, articles discussed the causes of childhood obesity in varying levels of detail. Individual causes were often cited, for instance unhealthy eating habits: “*If parents are not preparing fresh meals or are using lots of processed food then the problem just gets passed down to the younger generation*." In these quotes (also see Table 1), the responsibility is lain with the parents and the adolescent, although other articles question this assumption: “*XXX does not deny that parents can be responsible for their children's obesity, but he believes there are other significant factors.”*

Several articles also discussed societal factors linked to obesity, including poverty: “*[…] in Philadelphia, where roughly a quarter of residents live below the poverty line, 41% of children ages 6 to 17 are overweight or obese.”*

### Sparking moral outrage

Newspaper articles often contained negative discourse on obesity in adolescents and regarding surgery, or specifically attributed blame to adolescents or their parents.

#### Stigmatizing obesity

Language used to describe adolescents with obesity, irrespective of being considered for surgery, was often derogatory and stigmatizing: “*A severely obese teenager is likely known by every other student in the high school not because she is a prom queen, but because she is physically the largest student in the school.”* In this instance, there is a specific reference to the adolescents not meeting beauty norms, rather being socially isolated because of their weight. Other pejorative wording was also used, such as “*These are not kids who are pleasantly plump”*, invoking a contrast between adolescents who are severely obese and those who are merely ‘plump’.

A limited number of articles reacted explicitly against this dominant characterization, generally by referring to obesity as a disease that is beyond simple individual control:

*He speaks up now when someone speaks derisively of a person who is obese. He knows how it feels to be shunned for what medical researchers now deem a chronic disease, not a lifestyle choice. Without that experience, he said, I don’t think I would have that lens of compassion for people with their struggles.*

#### Who’s to blame?

As illustrated when describing the causes of obesity and stigmatization, some articles attributed blame to the adolescents and, particurarly, their parents:

*The two-part series called Junk Food Kids: Who's To Blame? features one 13-year-old schoolgirl who weighs 16st. XXX, who came to Britain from Romania four years ago, visits King's College Hospital in London with her parents to be assessed for the £12,000 surgery. During a consultation about the operation, XXX’s mother,[…]. Speaking in broken English, she explains: We keep on diet […].*

Here, the moral indignation about obesity is reinforced by including remarks that allude to xenophobic representations of non-UK born citizens, including the idea that newcomers profit from expensive NHS-payed surgery. The failure of the parents is underlined again: *“But just 15 min later, the family are shown heading straight to a nearby McDonald's for one last treat'."* Notably, the family themselves seemed to accept this blame: *“Her father YYY says: Dieting, changing your eating style, is about the power of willing. And we've demonstrated we're not that strong.”*

#### Surgery is a ‘quick fix’

A recurrent argument against bariatric surgery for adolescents was that it represents ‘choosing the easy way out’: *“Concerns have been raised that people are opting for stomach surgery as an 'easy option' rather than go to the trouble of changing their lifestyle, eating more healthily and taking exercise.”* While severe obesity was presented as a major problem, not all solutions were deemed acceptable, and losing weight through ‘hard work’ seemed to be valued more. This normative view was most explicitly expressed in the following:

*Young people should be given the chance to control their own weight, […]. Surgery takes it out of your hands. The message we are giving to young people, especially if their parents have had surgery, is that they don't really need to do anything, […], because it will be solved for them.*

This view is connected to the stigmatization of obesity, stressing that individual solutions displaying a ‘work ethic’ are required to solve a problem defined by a lack of this, in line with an implicit notion of laziness ascribed to people with obesity. Notably, articles arguing that surgery is not a quick fix similarly stressed the need for lifestyle changes to achieve success after surgery, possibly from the same assumptions about the character traits of the patients: "*Anyone considering weight loss surgery needs to understand that the surgeries are just a tool and they all require patients to make dietary changes and lifestyle modifications to be successful and to maintain weight loss. None of the operations are a quick fix”*.

### Sensation-seeking

This theme focuses on the style rather than the content. Sensationalist wording was frequently employed, which captured the attention of the reader and often contributed to a moralizing view on these topics. Notably, sensationalism was considerably more present in UK compared to US newspaper articles.

#### Headlines

Several articles featured headlines that set the tone for the article and contributed to a stigmatization of obesity in adolescents: *“Teens get gastric bands as obesity time-bomb explodes”* and *“100,000 TEENS NEED FAT OPS; SUPER-OBESE KIDS CRISIS”.* In other headlines, bariatric surgery was described as a potential solution, albeit a radical one: *"A Daunting Operation Offers Relief to Obese Teenagers".*

#### Sensationalism in personal stories

Several articles included a personal story of an adolescent with severe obesity who has undergone, or is contemplating surgery, which were sometimes written in an unnecessarily dramatic and sensational way: "*Two years earlier the fire brigade had had to demolish part of her parents' house in XXX, to take her to hospital.”* This amounted to a humiliation of this girl with the aim of capturing the readers’ imagination. Highlighting certain words or sentences in upper case also added to the sensationalist presentation of adolescent obesity: *“But XXX’s need to lose weight became frighteningly urgent last April when, aged just 19, she went BLIND. […] She was also at risk of developing a brain aneurysm, which meant she could have died at any moment.”* Interestingly, another article discussing this girl’s condition stated that she did not go blind, but rather had transient episodes of poor vision. She was at risk of vision loss because of intracranial hypertension. This implies that the earlier newspaper had overstated her medical condition for the sake of sensation.

#### ‘Radical’ surgery

Even in articles in which bariatric surgery was presented as a key therapeutic option, the radical or controversial nature of the procedure was emphasized: *“The schoolboy is one of an increasing number of youngsters who have had the controversial weight-loss operations, despite recommendations that such high-risk surgery should be a last resort […].”* The following description reinforced this through a vivid description: *“So she has come for a Roux-en-Y gastric bypass, the most radical treatment for obesity. It is, as one surgeon puts it, "a mutilating operation" in which a person's innards are rearranged with the aim of reducing eating. And it is booming in popularity.”*

### Raising ethical issues

#### Ability to give consent

Several ethical issues related to bariatric surgery were also discussed in the articles, including the question of consent by underage adolescents: “*[…] action must be taken to protect under-18 s, who are not mature enough to make a decision like this. Having a gastric band will affect the rest of your life."* One comment by a surgeon refers to the need for a multidisciplinary decision on whether to go ahead with surgery:

*Does he think a 14-year-old can give informed consent to have surgery which comes with long-term aftercare? No, I don't. I don't believe they can make properly judged consent over a long-term issue. […] With patients he has operated on at the younger end of the age range, I judged them to have capacity and the family were in agreement, and their psychologist was.*

This is crucial, given that adolescents frequently have unrealistic expectations, which need to be tempered: *“Although it has "definitely been worth it", she wasn't prepared for how difficult living with a gastric band would be. […] It is a lifelong commitment, which as a teenager she says she didn't really grasp.”*

#### Unmet need and disparity in access to care

Despite frequent negative views on obesity, and the criticism of surgery as a quick fix in some articles, most articles advocated for surgery as a tool to treat adolescents with severe obesity. One issue repeatedly identified in this context is the clinical unmet need and disparities in access, as only a fraction of eligible adolescents are being treated surgically: *“Recent data showed just 2,000 weight loss operations are carried out on children and teenagers each year, despite surgery rates having tripled in the last 20 years."* Because of the potential side effects, surgery is often not even presented as an option to patients:

*An estimated three to four million adolescents are heavy enough to meet the criteria for bariatric surgery, Dr. XXX said. But only about 1,000 teenagers a year have the operation. Many medical centers will not perform it on teenagers and many pediatricians never mention it to their heavy patients.*

Especially in the US articles, unequal access based on income and ethnicity was discussed: *“Childhood obesity disproportionately affects children of color and those in low-income populations, Dr. XXX said. Those getting access to surgery are almost exclusively middle- and upper-class white adolescents.”* A major additional barrier cited multiple times in the US context, was that both private and public insurers would not cover the surgery for underaged patients, contributing to the disparities in access.

#### Who should pay for surgery?

Although cost-effective, bariatric surgery is a relatively expensive treatment, and several articles connected the previous themes to the cost posed to the society: “"*A GIRL who weighs 25 stone at 16 is making a desperate effort to slim down with a £6,000 gastric bypass op on the NHS. […] Instead, she will have the surgery to stop her eating so much, with the bill picked up by the taxpayer*." In this extract, there is a direct link between stigmatization of obesity and indignation that the taxpayer, through the NHS, will have to pay for the treatment. A similar statement was quoted above in the section on blame. In one article, the question was directly posed to the readers: *“What do you think—SHOULD GASTRIC BANDS BE PAID FOR BY NHS?”* However, other articles stressing the unmet need for surgery argue that bariatric surgery is cost-effective: *“We know we should be doing more adult bariatric surgery. This is probably down to cost, he says, though surgery is cost effective to the health service in the long term."*

## Discussion

This qualitative study aimed to investigate the moral framing of adolescent bariatric surgery in UK and US news media using thematic analysis. The predominant themes identified related to (1) defining the burden of adolescent obesity, (2) sparking moral outrage, (3) sensation-seeking, and (4) raising ethical issues.

The first theme captured statistics on the prevalence of adolescent obesity, as well as the broader discussion on the causes of childhood obesity, which could be classified as person-level or system-level. The general public does not view these as mutually exclusive, as a UK study reported an agreement of approximately 60% with both ‘People are overweight because they lack willpower’ and ‘People are overweight because there are so many unhealthy foods around’ [23]. While both ways of causal reasoning were found in our dataset, a recent study found that discussions of social and economic aspects related to obesity in UK newspaper articles have decreased over time, with increasing emphasis placed on individual factors [24]. Moreover, concentrating on individual causes was connected to the subthemes of whom to attribute blame to, and to what extent the taxpayers should contribute to surgical treatment. The latter point is made more salient by the repeated description of bariatric surgery in adolescents as ‘radical’, a specific framing of the intervention that can contribute to moral outrage.

Personal stories, and even article headlines, contained non-neutral, vivid and often sensationalist terms, signaling a moral evaluation or judgment of obesity. Indeed, there was some evidence of disparaging descriptions of adolescents with severe obesity, by both journalists and HCPs. A focus on individual rather than systemic causes of obesity in news articles could contribute to the further stigmatization of people with obesity [25], including by HCPs [26]. Stigmatization of obesity is often societally tolerated on the belief that it will motivate those affected to ‘correct themselves’ and lose weight, yet research clearly indicates that stigmatization creates additional barriers to attain healthy lifestyles and thereby worsens obesity [27]. Bariatric surgery is often viewed negatively as a ‘quick fix’ option because not all weight-loss options are evaluated equally, in part because some do not conform to the societal norms of hard work and personal responsibility, or of being able to tackle your own problems with your own resources. And although scientific studies and HCPs were extensively quoted, often endorsing bariatric surgery to counter adolescent obesity, and stressing the gap between the number of eligible patients for surgery and the number of procedures performed, the frequent referrals to bariatric surgery as ‘radical’ or even ‘mutilating’ seem to reflect an ambivalence by journalists towards these recommendations [28].

Informed consent or assent was identified as a specific ethical challenge, with several articles raising the question whether and to what extent obese adolescents could give consent for surgery. Even in adults, and more so in adolescents, difficulties can arise when informing patients about potential complications, expected long-term benefits and mandatory changes to lifestyle [15]. Health illiteracy can be an additional obstacle in obese adolescents and their family. Furthermore, negative or stigmatizing reporting in the media might also impair patients’ autonomy [15] by affecting which treatments are viewed by patients and their families in their particular socio-economic context as feasible or even desirable.

Our finding that sensationalism (language used in news title and text) was more present in the UK articles (in our sample) is intriguing. One potential explanation for this is that most relevant US articles were published in local newspapers, in contrast to UK articles, published mainly in tabloid newspapers supportive of conservative ideology. In contrast, disparity in access to treatment, including surgery, is an important ethical theme [15] which featured most prominently in our analysis of US newspaper articles. Emphasizing this in media articles could help build support for policy initiatives to tackle this issue.

Summarizing the implications from our study to formulate recommendations for improving future discussions of adolescent obesity and bariatric surgery in the media, we would stress the need for both HCPs and journalists to be mindful of how the use of language from certain registers might frame the topic. Since HCPs are often interviewed, they can leverage this opportunity to stimulate a more constructive and well-balanced discussion by providing accurate information to journalists – although there is a paucity of literature on this topic – and thereby become actively involved in destigmatizing health issues [29]. In a similar vein, journalists who write about health topics should be aware of how they present their story, because this determines what the public, and indirectly the policymakers, will consider the facts to be. Framing the public discussion in a certain direction could preclude other views on the issue [18]. Interestingly, a study on a range of public health service messaging also found that these often focused on individual responsibility or perpetuated stereotypes, and concluded that by understanding the problems with these stereotypes, health communicators should be educated on developing alternative messaging that are less harmful [30]. A comparable approach for journalists, for instance through medical education workshops and the introduction of editorial policies with the aim of avoiding certain language, might improve the coverage in the future. Furthermore, policymakers should resist the sensationalist characterization of obesity, instead recognizing that childhood obesity is a complex issue without clear-cut or one-dimensional solutions. Lastly, as mentioned, the framing of obesity in the media might affect which treatments are viewed as acceptable by patients and their families, as well as the general public. Heightened awareness of this can assist HCPs to identify underlying preconceptions and misconceptions of patients and their family, which can be addressed when discussing and evaluating treatment options [31].

Our study has several limitations. First, as articles were searched for using one database, LexisUni, we cannot exclude that relevant articles were omitted because they were not included. It is unclear if the relatively limited number of articles retrieved from, mostly local, US newspapers, is partly the result of our search methodology or reflects a true underreporting compared to UK news media. These findings are mirrored in a 2022 news media survey, with a comparable usage of print and online news media for both countries, but with a bigger role for regional or local newspapers in the US [32]. In addition, we did not include televised news broadcasts or social media news articles in our analysis, which might report differently on adolescent bariatric surgery than classic newspaper articles.

## Conclusions

In conclusion, this study investigated the moral framing of adolescent bariatric surgery in newspaper articles, and identified implicit and explicit moral framing. Despite frequent citing of experts and studies on the efficacy, safety and unmet need of surgery, obesity and surgery in adolescents were often stigmatized and sensationalized with (prospective) patients depicted as looking for an easy way out in the form of a solution brought by others (HCPs and the public in the form of taxpayers), in contrast to one they would have to work for. Representing patients this way may contribute to the stigma surrounding bariatric surgery, particularly in adolescents.

## Supplementary Information

**Additional file 1:** **Supplementary Table 1.** Overview of included newspaper article metadata.

## Data Availability

The newspaper articles analyzed for this paper are referred to in Supplementary Table 1. Annotations of these articles in Nvivo are available from the corresponding author on reasonable request.
