# Correction to: ‘A radical operation’ – a thematic analysis of newspaper framing of bariatric surgery in adolescents

**DOI:** 10.1186/s12889-023-15575-1

**Published:** 2023-04-26

**Authors:** Sander Lefere, Kato Verghote, Ruth De Bruyne, Veerle Provoost, Priya P. Satalkar

**Affiliations:** 1 Hepatology Research Unit, Department Internal Medicine and Pediatrics, Ghent University, Ghent, Belgium; 2 Liver Research Center Ghent, Ghent University, Ghent, Belgium; 3 Department Moral Sciences and Empirical (Bio) Ethics Research, Bioethics Institute Ghent, Ghent University, Ghent, Belgium; 4 Pediatric Gastroenterology, Hepatology and Nutrition, Department Internal Medicine and Pediatrics, Ghent University, Ghent, Belgium

**Correction to**: ***BMC Public Health*23, 447 (2023)**

10.1186/s12889-023-15366-8

Following publication of the original article, the authors identified an error in the author name of Kato Verghote.

- The incorrect author name is: Kato Vergote.
- The correct author name is: Kato Verghote.

