# Supplementary material for: ‘A radical operation’ – a thematic analysis of newspaper framing of bariatric surgery in adolescents
Source: BMC Public Health. 2023 Mar 7;23:447. doi: 10.1186/s12889-023-15366-8 (PMC9993750; doi:10.1186/s12889-023-15366-8)
Supplement: Supplementary file 1 — Additional file 1: Supplementary Table 1. Overview of included newspaper article metadata. [file 12889_2023_15366_MOESM1_ESM.docx]

| Supplementary Table 1. Overview of included newspaper article metadata | | | |
| --- | --- | --- | --- |
| Year | Title | Newspaper | Word count |
| UK Newspaper articles | | | |
| 2014 | 13-YEAR-OLDS GIVEN GASTRIC BANDS; Expert warns that obesity will bankrupt the NHS | Sunday Express | 733 |
| 2015 | Number of teens receiving weight loss surgery soars | Birmingham Evening Mail | 515 |
| 2015 | NOW DOCTORS GIVE BOY, 12, GASTRIC BAND | Daily Mail | 1099 |
| 2015 | I had a gastric band fitted at just 13... and it nearly strangled me to death when I grew up; survivor calls to end weight-loss ops for kids | Daily Mirror | 1152 |
| 2015 | 140,000 fat UK kids need gut-staple op | Daily Star | 224 |
| 2015 | KIDS AGED13 GET OBESITY OPS ON NHS; Shock rise in children's fat-fighting | Daily Star | 440 |
| 2015 | Teens get gastric bands as obesity time-bomb explodes | Irish Independent | 527 |
| 2015 | 30 stone at 13: meet the obese teenagers going under the knife; It's drastic, dangerous and divides doctors - why are so many young Britons having gastric surgery? | The Guardian | 4071 |
| 2016 | I became so fat it made me blind; Teen's sight saved by op EXCLUSIVE | Daily Mirror | 762 |
| 2016 | A RECIPE FOR CHILD NEGLECT; Morbidly obese children are being taken into care. But are their parents really to blame? | Sunday Times | 3290 |
| 2016 | 25 STONE AGED 16; EXCLUSIVE: SAD PROOF UK'S FATTEST IN EUROPE Gastric band op to save Lucy's life 22st dad to get one at same time | The Sun | 929 |
| 2017 | Dublin hospital to begin gastric band operations on children | Belfast Telegraph | 172 |
| 2017 | HSE plans surgery for obese children | Irish Independent | 639 |
| 2017 | YOUNG AND FAT; nhs figures reveal that nearly half of youngsters are overweight or obese and risk an early grave | The Express | 1091 |
| 2017 | 100,000 TEENS NEED FAT OPS;SUPER-OBESE KIDS CRISIS | The Sun | 202 |
| 2018 | The NHS can help obese children; The Surgery DOCTOR'S DIARY | Daily Telegraph | 712 |
| 2018 | Weight-loss ops for teens need to rise 100-fold' | Daily Telegraph | 454 |
| 2018 | Before, I couldn't walk 200m'? - why surgery can be life-changing for super-obese children | The Guardian | 1521 |
| 2018 | FAT KIDS OP PLEA Overweight teenagers must have weight-loss surgery to stop 'obesity apocalypse', leading doctor claims | The Sun | 494 |
| 2019 | Teenager who weighed 275lbs undergoes weight loss surgery at SIXTEEN to avoid the same fate as her obese mother who died of a heart attack | Daily Mail | 809 |
| 2019 | Severely obese children as young as TWELVE should be offered gastric bypasses 'because it is safe and effective', paediatricians say | Daily Mail | 821 |
| 2019 | Call for more obese kids to have gastric bypass surgery | Daily Mirror | 432 |
| 2021 | Children need gastric bands to treat diabetes, says surgeon | Daily Telegraph | 410 |
| 2021 | Hundreds of fat children should be given NHS weight-loss ops to reverse their type 2 diabetes, leading medic claims | Daily Mail | 927 |
| 2021 | Give fat children weight loss surgery because it's safe and works, doctors say | Daily Mail | 885 |
| 2021 | The experts weigh in on how to beat our obesity crisis | Daily Mail | 2185 |
| US Newspaper articles | | | |
| 2015 | Study: Teen obesity surgery benefits last at least 3 years | Northern Illinois University | 797 |
| 2015 | Bariatric surgery in teens shows promise | Pittsburgh Tribune Review | 715 |
| 2017 | Doctors Consider a Last Best Hope for Obese Teenagers: Surgery | New York Times | 1974 |
| 2017 | Film carries RCHS student to Times Square | Rockwall County Herald-Banner | 833 |
| 2019 | Experts now say bariatric surgery is safe for teens with severe obesity. One from Aurora tried it. | Chicago Daily Herald | 1244 |
| 2019 | Kids with obesity: Get weight loss surgery? | Deseret Morning News | 2419 |
| 2019 | A Daunting Operation Offers Relief to Obese Teenagers | New York Times | 1162 |
| 2019 | Weight-Loss Surgery for Teens Who Can’t Lose Weight Any Other Way | New York Times | 1278 |
| 2019 | Bariatric surgery is safe for teenagers, Penn/CHOP study finds | Philadelphia Inquirer | 527 |
| 2019 | This Jefferson County teen has everything on track – except her weight. Now she hopes surgery can help. | St. Louis Post | 2499 |
| 2020 | For the young, a new start; Bariatric surgery is beneficial but rarely used for teens with severe obesity. | Philadelphia Inquirer | 1485 |
| 2022 | COVID's thumb on the scale; Docs: Kids' weight gain making some more vulnerable to virus | Philadelphia Daily News | 1368 |
